# Hyaluronan induces odontoblastic differentiation of dental pulp stem cells via CD44

**DOI:** 10.1186/s13287-016-0399-8

**Published:** 2016-09-20

**Authors:** Naoki Umemura, Emika Ohkoshi, Masamichi Tajima, Hirotaka Kikuchi, Tadashi Katayama, Hiroshi Sakagami

**Affiliations:** 1 Division of Pharmacology, Department of Diagnostic and Therapeutic Sciences, Meikai University School of Dentistry, 1-1 Keyakidai, Sakado, Saitama 350-0283 Japan; 2 Department of Oral Biochemistry, Asahi University School of Dentistry, Gifu, 501-0296 Japan; 3 Department of Natural Products Chemistry, Faculty of Pharmaceutical Science, Aomori University, Aomori, 030-0943 Japan; 4 Division of Endodontics, Department of Restorative and Biomaterials Sciences, Meikai University School of Dentistry, Saitama, 350-0283 Japan; 5 Division of Operative Dentistry, Department of Diagnostic and Therapeutic Sciences, Meikai University School of Dentistry, Saitama, 350-0283 Japan

**Keywords:** Dental pulp calcification, Bone mineralization, DMP-1 protein, DSPP protein, Dental pulp capping, Smad1 protein

## Abstract

**Background:**

Dental pulp tissue contains many undifferentiated mesenchymal cells, which retain the ability to differentiate into mature cells. Induced pluripotent stem cells have been developed from various cell sources, including dental pulp-derived stem cells, and evaluated for potential application to regenerative therapy. Dental pulp tissues overexpress CD44, a cell-adhesion factor involved in the induction of mineralization. In this study, we investigated the effects of hyaluronan—a known CD44 ligand—on dental pulp stem cells (DPSCs).

**Methods:**

DPSC CD44 expression was analyzed using immunofluorescence staining, flow cytometry, and western blotting. Cell proliferation was evaluated using the 3-(4,5-dimethylthiazol-2-yl)-2,5-diphenyltetrazolium bromide assay. Effects of hyaluronan on the cell cycle were analyzed by flow cytometry. Alkaline phosphatase activity was employed as marker of mineralization and measured by fluorometric quantification and western blotting. Bone morphogenetic protein (BMP)-2, BMP-4, dentin sialophosphoprotein (DSPP), and dentin matrix acidic phosphoprotein 1 (DMP-1) levels were measured using real-time polymerase chain reaction. Odontoblastic differentiation and the close cell signaling examination of DPSC differentiation were determined using western blotting.

**Results:**

Hyaluronan induced expression of the odontoblastic differentiation markers DMP-1 and DSPP. Moreover, the odontoblastic differentiation induced by hyaluronan was mediated by CD44—but not by Akt, Smad1 or MAPK signaling.

**Conclusions:**

Our results indicate that hyaluronan induces odontoblastic differentiation of DPSCs via CD44. This suggests that hyaluronan plays a crucial role in the induction of odontoblastic differentiation from DPSCs. Our findings may aid the development of new, inexpensive, and effective conservative treatments for dental pulp repair.

**Electronic supplementary material:**

The online version of this article (doi:10.1186/s13287-016-0399-8) contains supplementary material, which is available to authorized users.

## Background

Dental pulp cells have the capacity to differentiate into odontoblasts. Dental damage caused by oral cavities, periodontal disease, or mechanical trauma induces the formation of reparative dentin, a poorly organized mineralized matrix that serves as a protective barrier to the dental pulp [1].

Dental pulp stem cells (DPSCs) are present in human dental pulp, even in adult pulp, as clonogenic and highly proliferative cells obtained after enzymatic disaggregation [2]. These cells harbor the characteristics of plastic adherence and express stem cell markers such as CD29, CD90, CD44, and CD146 [2]. Additionally, DPSCs express transcription factors expressed by embryonic stem cells, including Oct-4, Sox-2 and Nanog [3, 4]. Numerous researchers have since shown that DPSCs retain the capacity for both self-renewal and multiple cell lineage differentiation [5, 6] and can be stimulated, under specific conditions, to differentiate into various cell types such as adipocytes, myoblasts, neurons, chondrocytes, odontoblasts and osteoblasts both in vitro and in vivo [7–9]. Animal studies have also revealed great potential for DPSCs in the repair and regeneration of various tissues, including bone [10], muscle [3] and teeth [11].

Odontoblasts, especially those in the root ends of immature teeth, express CD44, which is strongly expressed by cells undergoing mineralization, such as ameloblasts, odontoblasts and osteoblasts in calcifying areas [12]. CD44 functions as an adhesion molecule and is a broadly distributed type I transmembrane glycoprotein receptor for the glycosaminoglycan hyaluronan (HA) [13, 14]. However, the effects of HA stimulation of CD44 on DPSCs remain unknown. In this study, we investigated the effect of HA on DPSCs.

## Methods

### Reagents and cell culture

Hyaluronic acid sodium salt (CAS number 9067-32-7) was purchased from Nacalai Tesque Co. (Kyoto, Japan). This reagent was slowly dissolved in double-distilled water to a final concentration of 10 mg/mL (1 %). HA was further diluted in culture medium to required concentrations prior to use in cell culture experiments. Dulbecco’s modified Eagle’s medium (DMEM) was purchased from Invitrogen (Carlsbad, CA, USA). Fetal bovine serum (FBS) was purchased from Nichirei Bioscience (Tokyo, Japan).

Human DPSCs were obtained from AllCells LLC (Emeryville, CA, USA). Cell cultures were maintained in DMEM supplemented with 10 % FBS and antibiotics (100 U/mL penicillin and 100 μg/mL streptomycin) at 37 °C in a humidified atmosphere containing 5 % CO_2_. The passage numbers were limited at 2–5 to avoid cell deterioration.

### Immunofluorescence

Monolayers of DPSCs were cultured with DMEM containing 10 % FBS for 48 h in four-well covered glass chamber slides. After two washes with phosphate-buffered saline (PBS) containing 1 % bovine serum albumin (Sigma-Aldrich, St. Louis, MO, USA), cell surface Fc receptors were blocked with immunoglobulin G (IgG) (Santa Cruz Biotechnology Inc., Dallas, TX, USA) on ice for 15 min. The cells were then stained for 30 min at 37 °C with a 1:100 dilution of a fluorescein isothiocyanate (FITC)-conjugated anti-CD44 monoclonal antibody (BD Biosciences, Franklin Lakes, NJ, USA) or an isotype-matched FITC-conjugated IgG control antibody (BD Biosciences). After washing, the cells were analyzed using an ECLIPSE TS100-F microscope equipped with an Intensilight C-HGFIE illumination system (Nikon Co., Ltd., Tochigi, Japan). Digital images were processed with NIS Elements BR3.2 imaging software (Nikon Co., Ltd.) and Adobe Photoshop 7.0 (Adobe Systems, San Jose, CA, USA).

### Evaluation of cell growth using the MTT assay

DPSCs were seeded into 96-well microtiter plates at a density of 1 × 10^3^ cells/well and allowed to adhere for 24 h. Cell viability was assessed on a daily basis by addition of 5 μL of 3-(4,5-dimethylthiazol-2-yl)-2,5-diphenyltetrazolium bromide (MTT) using a Cell Proliferation Kit I (Roche Diagnostics, Mannheim, Germany), according to the manufacturer’s instructions. The number of viable cells was assessed by measuring the absorbance of the produced formazan crystals at 595 nm with a MultiSkan JX microplate reader and Ascent software (Thermo Labsystems, Vantaa, Finland). The measurement was performed once per day for 5 days. Cell growth was calculated relative to the value on the first day, which was set at 100 %.

### Quantification of alkaline phosphatase

DPSCs were seeded into 24-well plates at a density of 5 × 10^4^ cells/well, and incubated with HA (1–20 μg/mL) for 1 week. The cells were then harvested as cell lysates using a SensoLyte® FDP Alkaline Phosphatase Assay Kit (AnaSpec, San Jose, CA, USA), which uses a fluorogenic assay to determine alkaline phosphatase (ALP) activity. The assay was performed according to the manufacturer’s instructions, and fluorescence signals were measured with SpectraFluor plus XFluor4 software (Tecan Japan Co., Ltd., Kawasaki, Japan).

### Flow cytometry analysis

For analysis of CD44-positive cell surface antigen expression, untreated and HA-treated DPSCs were harvested by trypsinization, washed with PBS, centrifuged into cell pellets and resuspended in fluorescence-activated cell sorting (FACS) buffer (PBS containing 0.5 % bovine serum albumin). The cells were stained for 30 min at 4 °C with a FITC-conjugated anti-human CD44 antibody (BD Biosciences, San Jose, CA, USA) or an isotype-matched FITC-conjugated IgG control antibody (BD Biosciences). Flow cytometry was performed using an EPICS Altra flow cytometer (Beckman Coulter, Brea, CA, USA) and the data were analyzed using Expo-3 v1.2B software (Beckman Coulter).

For cell cycle analysis, the cell cycle distribution of cells was assayed after 48 h by using flow cytometry to measure the DNA content of nuclei labeled with PI according to the manufacturer’s instructions (BD Pharmingen, BD BioSciences). Data acquisition and analysis were performed using an EC800 flow cytometer (Sony Biotechnology, Tokyo, Japan) with EC800 analysis software (Sony Biotechnology).

### Immunoblot analysis

Whole-cell extracts from DPSCs were obtained using a lysis buffer (10× RIPA buffer; Cell Signaling Technology, Beverly, MA, USA) supplemented with 1 mM phenylmethanesulfonyl fluoride plus one tablet of protease inhibitor cocktail (Complete, ethylenediaminetetraacetic acid (EDTA)-free; Roche Diagnostics GmbH, Mannheim, Germany). Aliquots of cell lysates (50 μg protein) were separated by 8 % or 12 % sodium dodecyl sulfate polyacrylamide gel electrophoresis and electroblotted onto polyvinylidene difluoride membranes. The membranes were probed with primary antibodies, comprising anti-CD44 mouse monoclonal antibody, anti-phospho-Akt rabbit monoclonal antibody, anti-Akt rabbit monoclonal antibody, anti-phospho-GSK3β rabbit monoclonal antibody, anti-phospho-Smad1 rabbit monoclonal antibody, anti-Smad1 rabbit monoclonal antibody, anti-phospho-β-catenin (Ser552) rabbit monoclonal antibody, anti-phospho-β-catenin (Ser675) rabbit monoclonal antibody, anti-β-catenin rabbit monoclonal antibody, anti-phospho-p44/42 MAPK rabbit monoclonal antibody (all from Cell Signaling Technology, Danvers, MA, USA), anti-dentin sialophosphoprotein (DSPP) mouse monoclonal antibody (Santa Cruz Biotechnology Inc.), anti-dentin matrix protein-1 (DMP-1) rabbit polyclonal antibody, anti-ALP rabbit monoclonal antibody (both from Abcam PLC, Cambridge, UK), and anti-beta-actin antibody (Sigma-Aldrich) at the dilutions recommended by the manufacturers. Signals were detected using corresponding peroxidase-conjugated secondary antibodies (anti-rabbit IgG antibody or anti-mouse IgG antibody; Cell Signaling Technology), and signal bands were visualized by chemoluminescence (Clarity™ Western ECL substrate; Bio-Rad, Hercules, CA, USA). The membranes and images were developed with a ChemoDoc™ Imaging System (Bio-Rad).

### Real-time polymerase chain reaction

Total RNA was purified using Trizol reagent (Invitrogen Life Technologies, Carlsbad, CA, USA), and 600 ng of total RNA was used for reverse transcription with an iScript™ Advanced cDNA Synthesis Kit (Bio-Rad). For real-time polymerase chain reaction analysis, 1 μL of cDNA sample at 1:20 dilution, 1 μL each of forward and reverse primers (final, 500 nM), 7 μL of nuclease-free water, and 10 μL of SsoAdvanced SYBR Green Supermix (Bio-Rad) were used. The following primers were used: DMP-1 (GenBank ID: NM_004407.3) forward primer 5′-CCTGAGGATGAGAACAGCTCCA-3′ and reverse primer 5′-GATCTGCTGCTGTCTTGAGAGTCAC-3′; DSPP (GenBank ID: NM_014208.3) forward primer 5′-CCAGAGCAAGTCTGGTAACGGTAA-3′ and reverse primer 5′-GTCACTGCCTTCACTGTCACTGTC-3′; bone morphogenetic protein (BMP)-2 (GenBank ID: NM_001200.2) forward primer 5′-GGAACGGACATTCGGTCCT-3′ and reverse primer 5′-GGAAGCAGCAACGCTAGAAG-3′; BMP-4 (GenBank ID: NM_001202.3) forward primer 5′-TCACTGCAACCGTTCAGAGGTC-3′ and reverse primer 5′-CCAATCTTGAACAAACTTGCTGGA-3′; and GAPDH (GenBank ID: NM_002046.5) forward primer 5′-GCACCGTCAAGGCTGAGAAC-3′ and reverse primer 5′-TGGTGAAGACGCCAGTGGA-3′. Reaction conditions were one 5-min cycle at 95 °C, followed by 45 cycles of 95 °C for 10 s and 72 °C for 10 s. The reactions and relative quantification analyses were performed using a LightCycler 480 instrument (Roche Diagnostics, Indianapolis, IN, USA).

### Signal blocking assays

DMH-1, a Smad1/5 inhibitor and SCH772984, a novel ERK1/2-specific inhibitor were purchased from Selleckchem.com (http://www.selleckchem.com/). LY294002, an Akt inhibitor, was purchased from Cell Signaling Technology. An anti-CD44 monoclonal antibody (Clone A3D8; Sigma-Aldrich) was used in neutralization assays.

DPSCs were pretreated with the inhibitors or CD44-blocking antibody for 30 min before stimulation with HA (10 μg/mL). After 30 min of stimulation, the cells were harvested to investigate the inhibition of phosphorylation for several signaling molecules. After 24 h, the odontoblastic differentiation markers DMP-1 and DSPP were evaluated by immunoblotting.

### Statistical analysis

Data are presented as the mean ± SD and evaluated using one-way analysis of variance followed by Dunnett’s multiple comparison. Values of *P* < 0.05 were accepted as statistically significant.

## Results

### Expression of CD44 in DPSCs

**Fig. 1:**
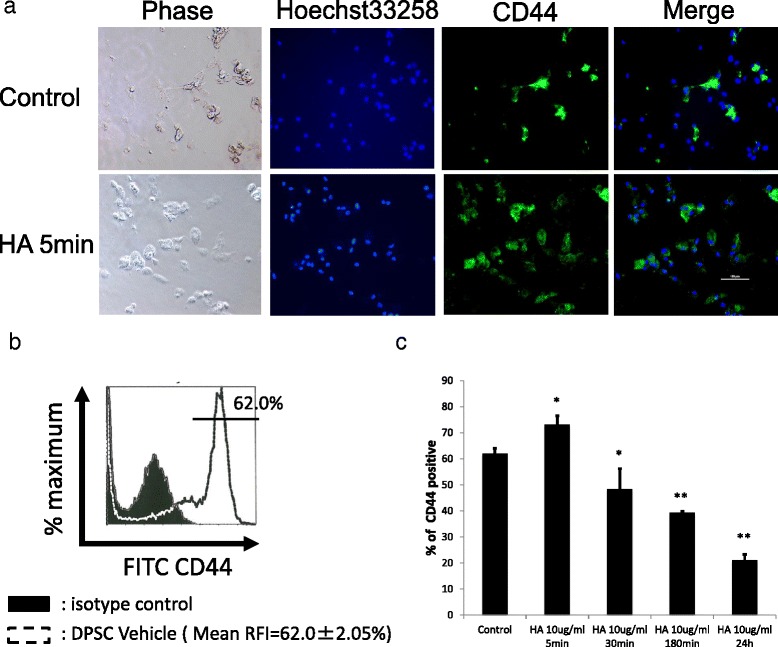
DPSCs express CD44 on the cell surface. **a** Live cell images showing signals from anti-CD44 antibody immunofluorescence (*green*) and Hoechst 33258 nuclear staining (*blue*). The rightmost panels indicate merged images from double staining. Scale bar: 100 μm. The *top panels* show DPSCs that were not treated with HA, while the *bottom panels* show DPSCs treated with HA at 10 μg/mL for 5 min. **b** Representative single-parameter diagrams showing CD44 expression by flow cytometry. *RFI* relative fluorescence intensity. **c** Bar graphs of the FACS analysis data for HA treatment time course of CD44 cell surface expression. Data are presented as the mean ± SD of three different experiments. ^*^ *P* < 0.05, vs. control. ^**^ *P* < 0.01, vs. control. *DPSCs* dental pulp stem cells, *FITC* fluorescein isothiocyanate, *HA* hyaluronic acid

Most CD44 antigenicity in dental pulp tissue is present in the incomplete region of the roots [12]. However, whether these CD44-expressing cells are DPSCs is unknown. Consequently, we identified CD44 expression on the DPSC cell surface (Fig. 1a, upper panels). Flow cytometry revealed a high proportion of CD44-positive cells (approximately 62 %; Fig. 1b, c). We then investigated whether CD44 expression was altered by treatment with HA, a known CD44 ligand [15]. We found that the number of CD44-expressing cells was significantly increased from 62 % to 72 % at 5 min following treatment with HA, while this expression significantly decreased to 54 % after 30 min and continued to decrease to 22 % (Fig. 1c).

### HA induces mineralization in DPSCs

**Fig. 2:**
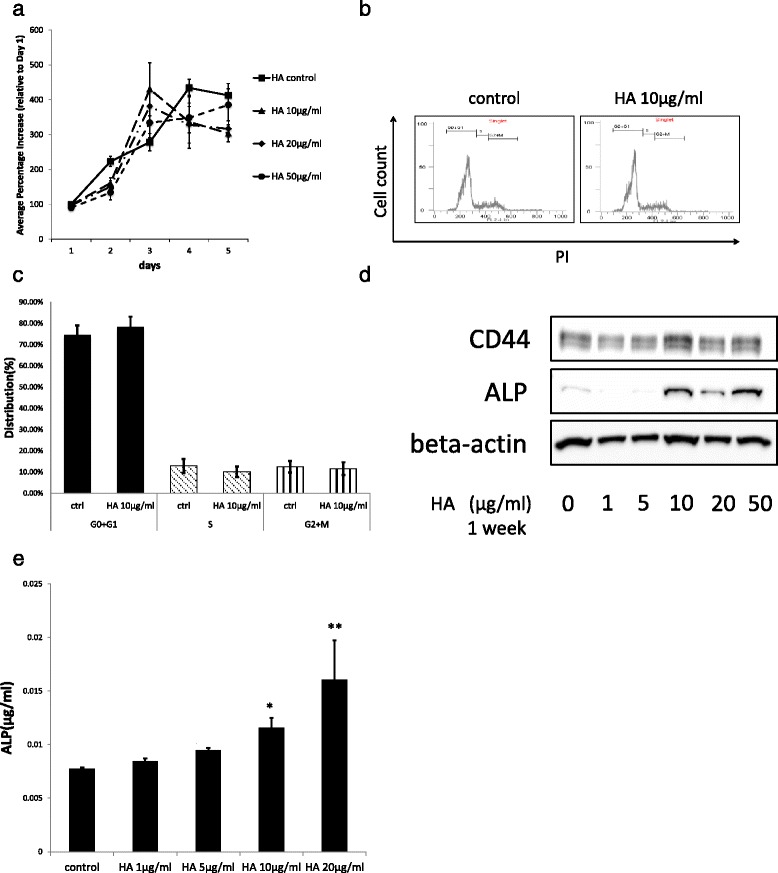
HA induces ALP, but does not influence dental pulp stem cell (DPSC) proliferation or cell cycle distribution. **a** DPSC growth curve following treatment with hyaluronic acid (HA). Increases are shown as percentages relative to the value on the first day of culture without HA, which was set at 100 %. Data are presented as the mean ± SD of at least three independent experiments. **b** Representative figures of cell cycle distribution are shown. DPSCs were treated with HA (10 μg/mL) for 48 h. Cell cycle distribution was analyzed by flow cytometry and cells classified into G0 + G1, S and G2 + M phases. **c** Bar graphs represent the percentage of cells within the different cell cycle phases of DPSCs treated as indicated. Data are presented as the mean ± SD. **d** ALP and CD44 protein levels were evaluated by western blotting. Whole-cell lysates of harvested DPSCs treated with HA at various concentrations for 1 week were examined. **e** Intracellular ALP concentrations were quantified using a fluorogenic ALP assay. Data are presented as the mean ± SD. ^*^ *P* < 0.05, vs. control. ^**^ *P* < 0.01, vs. control. *ALP* alkaline phosphatase, *HA* hyaluronic acid

We examined whether HA induces DPSC cell growth, and found that HA had no significant effect on the DPSC cell proliferation (Fig. 2a). Additionally, cell cycle analysis revealed that HA does not influence the cell cycle (Fig. 2b and c). We then investigated whether DPSCs were otherwise affected by treatment with HA, a known ligand of CD44. First, we considered whether DPSC mineralization was influenced by HA, because CD44 in pulp tissue plays an important role in mineralization [12]. Therefore, we used immunoblotting to measure ALP protein levels as an indicator of mineralization [6, 16]. ALP protein levels increased in a concentration-dependent manner when DPSCs were cultured with HA for 1 week (Fig. 2d and e). Next, we quantified the amount of ALP using a fluorogenic assay (Fig. 2e), and found similar results. These results suggest that DPSC mineralization was guided by HA.

### HA induces odontoblastic differentiation, but not osteogenic differentiation, in DPSCs

**Fig. 3:**
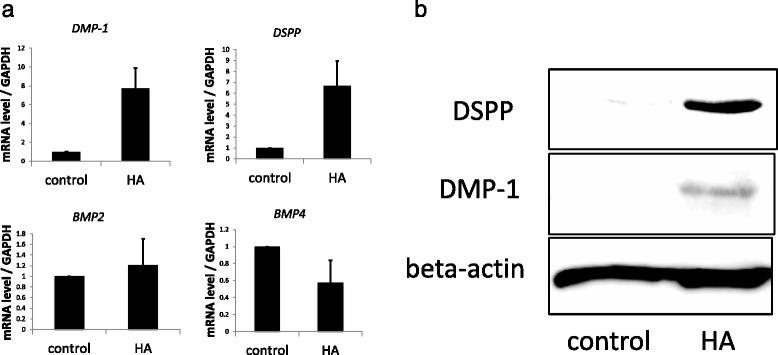
HA induces expression of DSPP and DMP-1 in DPSCs. **a** DPSCs were treated with HA (10 μg/mL) for 24 h, and then the mRNA levels of DMP-1, DSPP, BMP2, and BMP4 were measured using relative quantitative real-time PCR. **b** Protein levels of DSPP and DMP-1 were determined by western blotting. *BMP* bone morphogenetic protein, *DMP-1* dentin matrix protein-1, *DPSCs* dental pulp stem cells, *DSPP* dentin sialophosphoprotein, *HA* hyaluronic acid, *mRNA* messenger RNA

We also investigated how HA induces DPSCs to undergo mineralization, to evaluate whether HA induces odontoblastic differentiation or osteogenic differentiation. We measured the mRNA levels of BMP-2 and BMP-4 as osteogenic differentiation markers [17, 18] and DSPP and DMP-1 as odontoblastic differentiation markers [19, 20] in DPSCs cultured with HA for 24 h. We found that BMP-2 and BMP-4 mRNA levels underwent no significant changes, while DSPP and DMP-1 mRNA levels were markedly increased. The DMP-1 mRNA level increased 7.7-fold, while that of DSPP increased 6.7-fold (Fig. 3a). Additionally, we found that HA also increased DMP-1 and DSPP protein levels (Fig. 3b). These results suggest that HA stimulated DPSCs toward odontoblastic differentiation.

### HA-induced odontoblastic differentiation does not involve Akt, Smad1 or MAPK signaling

**Fig. 4:**
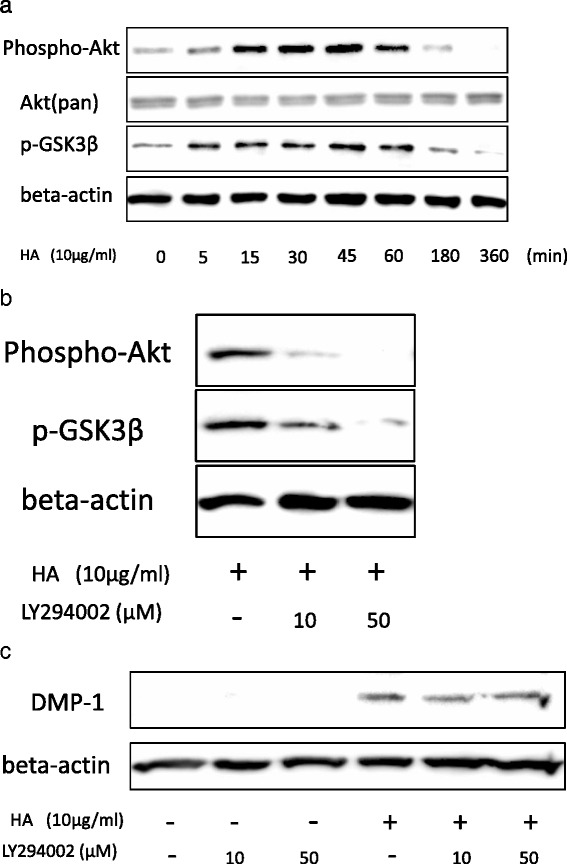
HA induces phosphorylation of Akt in DPSCs. **a** DPSCs were treated with HA (10 μg/mL) over a time course of 5–360 min, and then examined for phosphorylation of Akt and GSK3β by western blotting. **b** DPSCs were treated with HA (10 μg/mL) in the presence of LY294002 for 30 min, and the inhibition of Akt phosphorylation was validated. **c** Following inhibition of Akt phosphorylation by LY294002 treatment, DMP-1 expression was determined by western blotting. *DMP-1* dentin matrix protein-1, *HA* hyaluronic acid

**Fig. 5:**
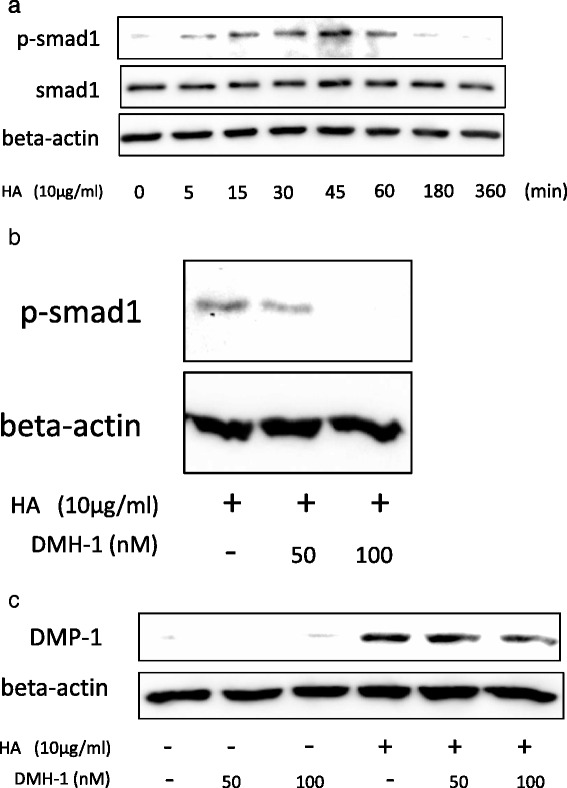
HA induces phosphorylation of Smad1 on DPSCs. **a** DPSCs were treated with HA (10 μg/mL) over a time course of 5–360 min and then examined for phosphorylation of Smad1 by western blotting. **b** DPSCs were treated with HA (10 μg/mL) in the presence of DMH-1 for 30 min, and the inhibition of Smad1 phosphorylation was verified. **c** Following inhibition of Smad1 phosphorylation by DMH-1 treatment, DMP-1 expression was determined by western blotting. *DMP-1* dentin matrix protein-1, *HA* hyaluronic acid

The above data suggest that HA stimulates DPSCs toward odontoblastic differentiation, but the underlying mechanism remains unclear. There have been few studies of signaling in DPSCs, so we next examined the types of intracellular signaling induced by HA in DPSCs on the basis of citations provided in a previous report [21]. We investigated whether HA could induce Akt phosphorylation, because the induction of differentiation by HA is thought to occur by activation of PI3 kinase and Akt in DPSCs [22, 23]. We found that peak levels of Akt and GSK3β phosphorylation occurred 30–45 min after treatment with HA (Fig. 4a), confirming that Akt signaling and downstream GSK3β signaling were activated in DPSCs. We then verified that DPSCs were induced to undergo differentiation into odontoblasts in response to this Akt signaling by examining whether the odontoblastic differentiation of DPSCs induced by HA stimulation was inhibited in the presence of LY294002, an Akt activation inhibitor [24]. We found that DMP-1 expression was not inhibited, although LY294002 did inhibit the Akt and GSK3β phosphorylation induced by HA treatment (Fig. 4b, c). We also investigated Smad signaling, as these signals are involved in osteogenic differentiation. Specifically, we examined whether HA could induce Smad signaling during odontoblastic differentiation of DPSCs. Smad1 phosphorylation reached a peak after 30–45 min of treatment (Fig. 5a), similar to findings for Akt. Furthermore, we investigated whether the odontoblastic differentiation mediated by HA could be inhibited by culture with DMH-1, a Smad1 phosphorylation inhibitor [25]. Although DMH-1 inhibited Smad1 phosphorylation after treatment with HA (Fig. 5b), DMP-1 expression was not inhibited (Fig. 5c).

**Fig. 6:**
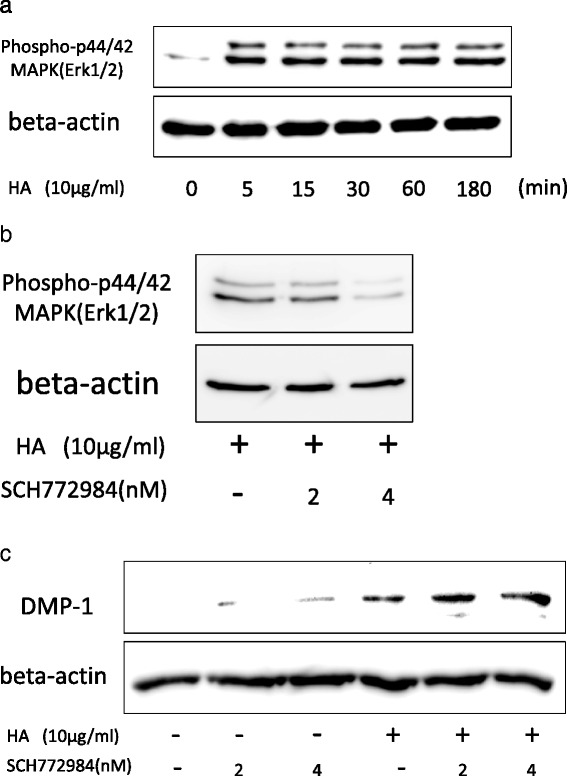
HA induces phosphorylation of Erk1/2 on DPSCs. **a** DPSCs were treated with HA (10 μg/mL) over a time course of 5–180 min and then examined for phosphorylation of Erk1/2 by western blotting. **b** DPSCs were treated with HA (10 μg/mL) in the presence of SCH772984 for 30 min, and the inhibition of Erk1/2 phosphorylation was verified. **c** Following inhibition of Erk1/2 phosphorylation by SCH772984 treatment, DMP-1 expression was determined by western blotting. *DMP-1* dentin matrix protein-1, *HA* hyaluronic acid, *MAPK* mitogen-activated protein kinase

Others have reported that HA-CD44 signaling in other cells activates Erk1/2, a mitogen-activated protein kinase (MAPK) [26, 27]. Therefore, we next investigated MAPK signaling. HA-induced DMP-1 expression was not affected by the inhibition of Erk1/2 phosphorylation by SCH772984, a novel inhibitor of Erk1/2 activation [28] (Fig. 6a–c). We also considered Wnt/beta-catenin signaling, because others have investigated the importance of this signaling pathway in odontogenic differentiation in recent years [29]. However, HA did not induce phosphorylation of beta-catenin (Additional file 1: Figure S1). These findings indicate that although Smad, Akt and MAPK signaling were all activated by HA in DPSCs, these pathways were not involved with the odontoblastic differentiation of DPSCs.

### Odontoblastic differentiation of DPSCs induced by HA treatment involves CD44 signaling

**Fig. 7:**
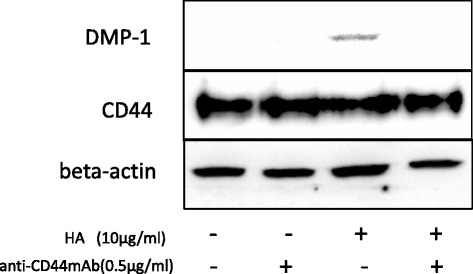
HA induces DMP-1 via CD44. DPSCs were treated with an anti-CD44 mouse monoclonal antibody to block CD44, and then examined for the inhibition of DMP-1 expression induced by HA (10 μg/mL). *DMP-1* dentin matrix protein-1, *HA* hyaluronic acid

Because HA is a known ligand of CD44, we investigated whether HA-mediated DMP-1 expression and odontoblastic differentiation arose via CD44 signaling. We precultured DPSCs with a CD44-neutralizing monoclonal antibody to inactivate CD44, and evaluated HA-induced DMP-1 expression levels. Inactivation of CD44 in DPSCs inhibited HA-induced DMP-1 expression (Fig. 7). This indicates that DMP-1 expression induced by HA involves CD44 signaling.

## Discussion

We initially aimed to investigate the type of differentiation induced by CD44 stimulation in DPSCs. Our data show that CD44 was expressed in approximately 62 % of DPSCs and that odontoblastic differentiation was promoted by HA-induced stimulation of CD44 in DPSCs.

Previous studies have suggested that DPSCs have the potential to regenerate dental tissues [30], myoideum and nerve tissues [9, 31]. Although dental pulp-derived induced pluripotent stem cells have been evaluated for use in clinical applications [32, 33], and HA stimulation of CD44 is important for differentiation leading to the production of odontoblasts, few studies have investigated the precise signaling mechanisms operating in DPSCs.

Low molecular weight HA can induce cell proliferation and induce osteocalcin mRNA expression in a dose-dependent manner in calvarial-derived mesenchymal cells [34]. However, others have reported that high molecular weight HA can induce mineralization of dental pulp tissue and dental pulp cells [35, 36]. Our investigation employed a high molecular weight HA. Treatment with this high molecular weight HA increased the proportion of CD44-positive DPSCs from 62 % to 72 % at 5 min posttreatment. Thereafter, cell surface expression of CD44 declined to 22 % at 24 h posttreatment (Fig. 1c). Meanwhile, CD44 levels in whole cell lysate remained unchanged following treatment with HA for either 24 h or 1 week (Fig. 2d and Fig. 7). These findings suggest that CD44 might shift into cells and away from the cell surface following HA treatment.

Although HA does not induce cell proliferation or affect the cell cycle in DPSCs (Fig. 2), we clearly demonstrated that HA signaling via CD44 is important for odontoblastic differentiation in DPSCs. Interestingly, HA induced activation of Smad1, Akt and Erk1/2, but not beta-catenin. The peak of Akt and Smad1 phosphorylation occurred 30 min after treatment with HA, while the peak of Erk1/2 phosphorylation occurred 5 min after HA treatment. Furthermore, degradation of phosphorylated Erk1/2 did not occur as was the case for Akt and Smad1 phosphorylation (Figs. 4, 5, and 6). However, while HA-induced Erk1/2 activation exhibited different characteristics to HA-induced Akt and Smad1 activation, these signaling pathways did not directly promote odontoblastic differentiation in DPSCs. This suggests that there may be another as-yet unexplained signaling mechanism by which HA induces odontoblastic differentiation via CD44. Therefore, our present results and those of previous studies on HA treatment and dental pulp [35, 36] suggest that HA induces odontoblastic differentiation via CD44 signaling in DPSCs. Our findings indicate that the application of HA to dental pulp medicine may be useful for dental pulp capping or tooth regeneration in the future. Despite our best efforts, the mechanisms underlying CD44-induced differentiation of DPSCs to odontoblasts remain unclear, and will require further examination in future studies. Nevertheless, our present study describes an efficient differentiation method to derive odontoblasts from DPSCs.

## Conclusions

HA induces odontoblastic differentiation of DPSCs via CD44, but does not promote cellular proliferation. While HA activates Akt, Smad and MAPK signaling, there is no clear relationship between these signaling pathways and the odontoblastic differentiation of DPSCs. These novel findings further our understanding of DPSC differentiation, and may facilitate advances in dental pulp therapy by enabling efficient induction of odontoblastic differentiation of DPSCs.

## Additional file

Additional file 1: Figure S1. HA does not induce phosphorylation of beta-catenin on DPSCs. DPSCs were treated with HA (10 μg/mL) over a time course of 5–360 min and then examined for phosphorylation of beta-catenin by western blotting. (PPTX 1259 kb)

## Abbreviations

ALP: alkaline phosphatase
BMP: bone morphogenetic protein
DMEM: Dulbecco’s modified Eagle’s medium
DMP-1: dentin matrix protein-1
DPSCs: dental pulp stem cells
DSPP: dentin sialophosphoprotein
EDTA: ethylenediaminetetraacetic acid
FACS: fluorescence-activated cell sorting
FBS: fetal bovine serum
FITC: fluorescein isothiocyanate
HA: hyaluronic acid
IgG: immunoglobulin G
MAPK: mitogen-activated protein kinase
MTT: 3-(4,5-dimethylthiazol-2-yl)-2,5-diphenyltetrazolium bromide
PBS: phosphate-buffered saline
PMSF: phenylmethanesulfonyl fluoride
