# Supplementary figures and images for: Hyaluronan induces odontoblastic differentiation of dental pulp stem cells via CD44
Source: Stem Cell Res Ther. 2016 Sep 20;7:135. doi: 10.1186/s13287-016-0399-8 (PMC5029108; doi:10.1186/s13287-016-0399-8)

## Slide 1
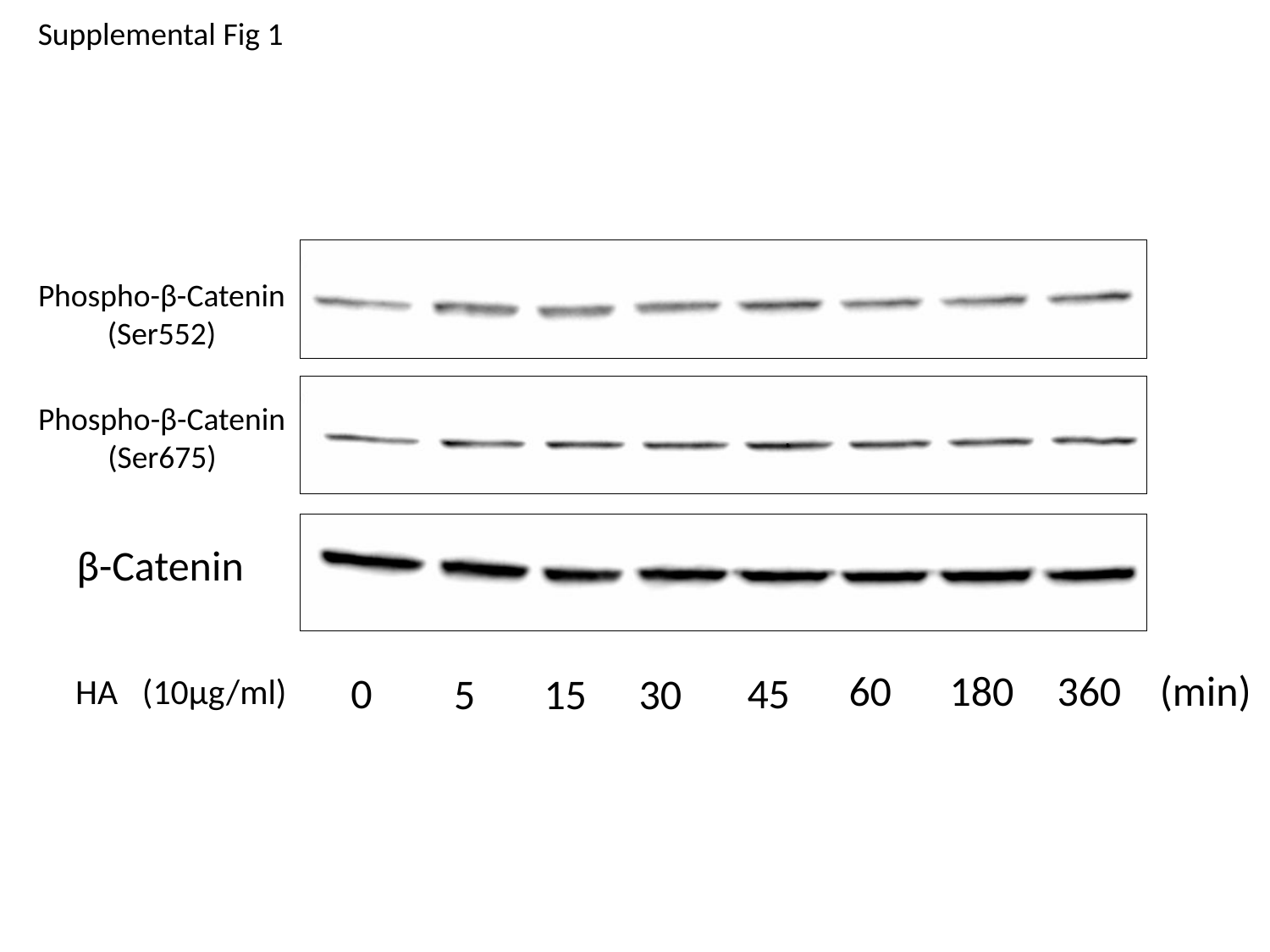

Supplemental Fig 1
Phospho-β-Catenin
(Ser552)
Phospho-β-Catenin
(Ser675)
β-Catenin
60
180
360
(min)
45
0
5
15
30
HA (10μg/ml)

Supplement: Additional file 1: Figure S1. — HA does not induce phosphorylation of beta-catenin on DPSCs. DPSCs were treated with HA (10 μg/mL) over a time course of 5–360 min and then examined for phosphorylation of beta-catenin by western blotting. (PPTX 1259 kb) [file 13287_2016_399_MOESM1_ESM.pptx]
